# Deciphering Fatty Acid Synthase Inhibition-Triggered Metabolic Flexibility in Prostate Cancer Cells through Untargeted Metabolomics

**DOI:** 10.3390/cells9112447

**Published:** 2020-11-10

**Authors:** Ju Eun Oh, Byung Hwa Jung, Jinyoung Park, Soosung Kang, Hyunbeom Lee

**Affiliations:** 1 Molecular Recognition Research Center, Korea Institute of Science and Technology, Seoul 02792, Korea; dhwndsm@naver.com (J.E.O.); jbhluck@kist.re.kr (B.H.J.); jypark@kist.re.kr (J.P.); 2 College of Pharmacy and Graduate School of Pharmaceutical Sciences, Ewha Womans University, Seoul 03760, Korea; sskang@ewha.ac.kr; 3 Division of Bio-Medical Science &Technology, KIST School, Korea University of Science and Technology, Seoul 02792, Korea; 4 Department of HY-KIST Bio-convergence, Hanyang University, Seoul 04763, Korea

**Keywords:** fatty acid synthase, metabolomics, metabolic flexibility, enzyme inhibition, glycerophospholipid metabolism

## Abstract

Fatty acid synthase (FAS) is a key enzyme involved in de novo lipogenesis that produces lipids that are necessary for cell growth and signal transduction, and it is known to be overexpressed, especially in cancer cells. Although lipid metabolism alteration is an important metabolic phenotype in cancer cells, the development of drugs targeting FAS to block lipid synthesis is hampered by the characteristics of cancer cells with metabolic flexibility leading to rapid adaptation and resistance. Therefore, to confirm the metabolic alterations at the cellular level during FAS inhibition, we treated LNCaP-LN3 prostate cancer cells with FAS inhibitors (Fasnall, GSK2194069, and TVB-3166). With untargeted metabolomics, we observed significant changes in a total of 56 metabolites in the drug-treated groups. Among the altered metabolites, 28 metabolites were significantly changed in all of the drug-treated groups. To our surprise, despite the inhibition of FAS, which is involved in palmitate production, the cells increase their fatty acids and glycerophospholipids contents endogenously. Also, some of the notable changes in the metabolic pathways include polyamine metabolism and energy metabolism. This is the first study to compare and elucidate the effect of FAS inhibition on cellular metabolic flexibility using three different FAS inhibitors through metabolomics. We believe that our results may provide key data for the development of future FAS-targeting drugs.

## 1. Introduction

Fatty acid synthase (FAS) is a multi-enzyme complex that synthesizes palmitic acid, a saturated fatty acid, through the condensation reaction of acetyl-CoA and malonyl-CoA, and it is a key enzyme involved in de novo lipogenesis. FAS is composed of two identical multifunctional monomers, each including seven different catalytic domains [1]. Lipids play a very important role in tumor cells and are involved in membrane biosynthesis, protein modification, and cell proliferation. Also, lipids are essential for lipid raft composition, generating lipid signaling molecules and supplying them to the oncogenic signaling pathway [2,3,4]. FAS is overexpressed in advanced cancers, while it is expressed at low levels in normal tissues due to a strict downregulation by the intake of fats and hormones [5]. It has been reported to be highly expressed in various cancers, such as breast cancer, colorectal cancer, stomach cancer, thyroid cancer, prostate cancer, ovarian cancer, and endometrial cancer [6,7,8,9,10]. Thus, FAS has become a novel therapeutic target molecule for cancer therapy [11].

Although FAS inhibitors have been developed steadily since the 1990s, first-generation inhibitors such as cerulenin have led to unexpected side effects [12]. Since then, many second-generation FAS inhibitors have been developed, and thus far, only TVB-2640 has entered the clinical trial stage. The other FAS inhibitors failed to enter the clinical trial stage because their FAS inhibitory effect in vitro did not correlate with the anticancer effect in animal models.

Several inhibitors of FAS have been shown to induce apoptosis in cancer cells and to cause tumor-growth delays in a cancer xenograft model. However, the mechanism is not well understood. Drug selectivity and pharmacologic limitations, especially metabolic limitations, have become a problem in preclinical and clinical settings of most FAS inhibitors [11,12]. Metabolic flexibility refers to cell adaptability in a new environment through metabolic change or reprogramming [13]. Cancer cells grow in a microenvironment through disturbing the homeostasis of self-regenerating cells. Aerobic glycolysis effectively represents the effect of the metabolic flexibility of the cancer cells, where it leads to an abrupt adaptation and resistance to the new microenvironment [14,15,16]. Thus, metabolic flexibility is important for cancer cells to survive. Additionally, it has been reported that when cancer cells are treated with drugs, metabolic flexibility is triggered [13,14,17]. In a similar context, it was found that anti-cancer potency through FAS inhibition is not effective due to possible metabolic flexibility. However, the underlying mechanism is still unclear [12]. 

Metabolomics is a type of ‘omics’ technology that includes a comprehensive characterization of metabolic products and metabolism, including the end products of cellular regulation processes in living systems [18]. Metabolomics improves understanding of disease pathology and therapeutic strategies for diseases with unmet challenges by identifying root causes [19]. To understand disease mechanisms, identify new drug targets, and in cases where clinical trial results are unexpected, metabolomics has become an important tool [20,21,22].

We hypothesize that when FAS is inhibited and de novo lipogenesis is blocked, there may be changes in the endogenous metabolism to compensate for the effect. Therefore, we believe that metabolic changes during FAS inhibition may provide key information for understanding metabolic flexibility in cancer cells. To our knowledge, there is no metabolism study of prostate cancer cells during FAS inhibition by inhibitors using metabolomics.

**Figure 1:**
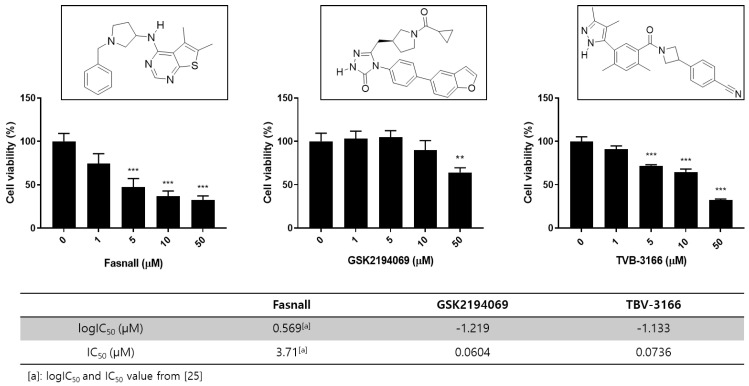
Overview of the chemical structures and cell viability after treatment with FAS inhibitors. Effect of FAS inhibitor treatment on LNCaP-LN3 human prostate cancer cell viability. The LNCaP-LN3 human prostate cancer cells were treated with different concentrations of Fasnall, GSK2194069, or TVB-3166 for 24 h, and cell viability was assayed using the WST-1 assay (mean ± SEM) and FAS inhibition assay with FAS inhibitors. The IC50 values of GSK2194069 and TVB-3166 were measured. (*** *p* < 0.001, ** *p* < 0.01).

Three FAS inhibitors were used for the metabolomics studies: Fasnall, GSK2194069, and TVB-3166 (Figure 1). TVB-3166 is a reversible and selective imidazopyridine-based FAS inhibitor. It has been reported that it causes a change in the cell signaling pathway that destroys the lipid raft structure and induces apoptosis [23]. GSK2194069 is a FAS inhibitor that selectively targets the ketoacyl reductase domain that competes with the substrate intermediates [24]. Fasnall is a recently developed potent inhibitor that targets multiple domains of FAS—namely, ketoacyl reductase (KR), enoyl reductase (ER), and malonyl/acetyltransferase (MAT)—and it is known to strongly and selectively inhibit FAS [25].

Here, we report the metabolic changes in the prostate cancer cell line LNCaP-LN3 after treating the cells with three different FAS inhibitors. Furthermore, we show the common metabolic changes in the cells after drug treatment and the metabolic changes that are specific to each inhibitor.

## 2. Materials and Methods

### 2.1. Materials

LNCaP-LN3 cells were obtained from KCLB (Korean Cell Line Bank, Seoul, Korea), and the following materials were used: RPMI medium (Gibco, Thermo Fisher Sceintific, Waltham, MA, USA), fetal bovine serum (Gibco), 100 U/mL penicillin (Invitrogen, Thermo Fisher Scientific, Waltham, MA, USA), 100 ug/mL streptomycin (Invitrogen), Fasnall (Cayman Chemical, Ann Arbor, MI, USA), GSK2194069 (Cayman), and solvents and chemicals including TVB-3166, and cell proliferation reagent WST-1, CCK-8, bovine serum albumin, palmitic acid-d31 were obtained from Sigma-Aldrich unless otherwise stated. Autoclaved Milli-Q water was used throughout the experiment.

### 2.2. Cell Culture

The human prostate cancer LNCaP-LN3 cells were obtained from the KCLB (Korean Cell Line Bank, Seoul, Korea) and maintained in RPMI medium (Gibco Life Technologies) supplemented with 10% fetal bovine serum (Thermo Fisher Scientific) and 1% antibiotics (100 U/mL penicillin and 100 ug/mL streptomycin, Invitrogen) in a humidified incubator at 37 °C and 5% CO_2._ Confluent cells were harvested by trypsinization and subcultured. The medium was changed every 3 days.

### 2.3. Cell Viability Assay

LNCaP-LN3 (7500 cells/well) were seeded in 96-well plates with 10% FBS and 1% antibiotics in RPMI medium with a total volume of 100 μL. After 24 h, cells were treated with different concentrations of Fasnall, GSK2496069, or TVB-3166 for 24 h. 10 μL of WST-1 reagent was added to the medium and incubated for 2 h. Then the absorbance was measured at 470 nm using a Versa Max 96-well plate reading spectrophotometer (Molecular Devices, San Jose, CA, USA).

For exogenous palmitate experiment, LNCaP-LN3 (7500 cells/well) were seeded in 96-well plates with 10% fatty acid-free BSA and 1% antibiotics in RPMI medium with a total volume of 100 μL. After 24 h, cells were treated with 50 μM of TVB-3166, with or without palmitic acid (100 μM or 200 μM). After the treatment and incubated for 24 h, 10 μL of CCK-8 reagent was added to the medium. After incubation for 2 h in a CCK-8 reagent-treated medium, the absorbance was measured at 505 nm using a Versa Max 96-well plate reading spectrophotometer (Molecular Devices, San Jose, CA, USA).

### 2.4. FAS Activity Assay

FAS lysate was obtained after performing three cycles of freeze-thawing of the LNCaP-LN3 cells. Enzyme, substrate, phosphate buffer, and inhibitor solutions in the inhibition kinetic assay were pipetted into the 96-well plate and conditioned for 30 min at 37. FAS inhibitors were pre-incubated with phosphate buffer, then substrates were added (0.4 mM malonyl-CoA, 0.24 mM acetyl-CoA, 0.5 mM NADPH) in a total reaction volume of 100 μL. The reaction was initiated by adding 40 μL of FAS. Dimethyl sulfoxide was used as a positive control and enzyme-free buffer was used as a negative control.

### 2.5. Metabolomics Study of FAS Inhibition

LNCaP-LN3 (0.6 × 10^6^ cells/well) were seeded in 6-well plates with 10% FBS and 1% antibiotics in RPMI medium. After 24 h, cells were treated with 50 μM of Fasnall, GSK2496069, or TVB-3166 (n = 5). After 24 h of incubation, cells were harvested using Corning cell scrappers. Ice-cold 70% methanol (100 μL) containing an internal standard (reserpine at a final concentration of 2 ppm) was added to the cell pellets, and the solution was vortexed for 30 s. Cells were lysed by three consecutive freeze/thaw cycles using liquid nitrogen, and the lysate was centrifuged for 10 min at 20,817× *g* (14,000 rpm). The 10 μL supernatant was immediately injected into a UPLC-Orbitrap-MS (ultra-performance liquid chromatography-orbitrap-mass spectrometry) system. A quality control (QC) sample, prepared by pooling equal volumes of each sample, was used for column conditioning, performed by injecting 10 times before the analytical runs. The QC sample was also analyzed after every 10 analytical sample runs to evaluate the repeatability of the instrument. The lysate supernatant was subsequently used for DNA normalization. DNA concentrations were analyzed using a nano-MD UV–vis spectrophotometer (Scinco, Seoul, Korea). 

### 2.6. Metabolomics Study of FAS Inhibition in Fatty Acid Free Condition

LNCaP-LN3 (0.6 × 10^6^ cells/well) were seeded in 6-well plates with 10% fatty acid-free BSA and 1% antibiotics in RPMI medium. After 24 h, cells were treated accordingly to their respective groups. D group was treated with 50 μM of TVB-3166, D + P group was treated with 50 μM of TVB-3166 and 200 μM of palmitate for 24 h. BSA-palmitate conjugation was performed as in a previous study [26]. Briefly, 200 mM palmitate was mixed with a 10% fatty acid-free BSA solution and conjugated at 37 °C for 2 h. The prepared stock solution of 2 mM BSA-palmitate was then mixed with serum-free RPMI media at a 1:10 ratio to form the medium containing 200 μM of BSA-palmitate. We have confirmed the absorption of the external palmitic acid into the cells by detecting the deuterated palmitic acid metabolite in the cells using UPLC-Orbitrap-MS. The metabolite was only found in the D + P group, indicating that the palmitic acid was successfully absorbed into the cells (Figure S3). The following method of cell harvesting and sample preparation for metabolomics analysis is identical to the method in Section 2.5.

### 2.7. Oil-Red O Staining

LNCaP-LN3 cells (0.6 × 10^6^ cells) were seeded in 35 mm dish with 10% fatty acid-free BSA and 1% antibiotics in RPMI medium. After 24 h, cells were treated with 50 μM of TVB-3166 for 24 h. Cells were washed twice with PBS and fixed on dishes with 4% paraformaldehyde solution for 30 min. Dried cells were stained with Oil-Red O solution for 30 min, followed by rinsing in DW and 60% isopropanol to remove unbound dye. For quantification of lipid accumulation, Oil-Red O was extracted with 100% isopropanol, and the optical density of the solution was detected at 490 nm. 

### 2.8. LC-MS/MS Instrumentation

Ultimate 3000 UHPLC linked to LTQ Orbitrap Velos Pro™ mass spectrometer (Thermo Scientific, San Jose, CA, USA) equipped with a heated electrospray ionization source was used. Together with the abovementioned systems, an Acquity^®^ UPLC HSS T3 column (1.8 μm particle size, 2.1 × 100 mm, Waters, USA) at 40 °C was used. For gradient elution, we have employed our method that was previously reported [27]. In brief, mobile phase A (0.1% formic acid in Milli-Q water) and mobile phase B (0.1% formic acid in methanol) at a flow rate of 0.4 mL·min^−1^ was used with the following gradient program: the initial conditions, 99% A and 1% B (*v*/*v*), were maintained for 1 min, and a linear gradient was initiated to reach 20% B over 2 min. A fast increase to 70% B for 4 min was then performed, and then mobile phase B was slowly increased to 100% over 6 min and maintained at 100% B for 2.5 min. The column was re-equilibrated to the initial conditions within 1.5 min and stabilized for 2 min. An additional QC sample was also analyzed after every 10 sample runs to validate the instrument conditions. The total running time was 18 min. All samples were maintained at 7 °C during the analysis, and the injection volume was 10 μL. For the detection of the metabolites, a mass-spectrometer with heated electrospray ionization source (HESI) in positive ionization mode was used with the following parameters: heater temperature, 200 °C; sheath gas flow rate, 35 arb (arbitrary units); auxiliary gas flow rate, 5 arb; sweep gas flow rate, 10 arb; capillary temperature, 320 °C; and S-lens RF level, 67.5%. The Xcalibur 2.2 (Thermo Scientific, San Jose, CA, USA) software system was used for data acquisition and processing. During data acquisition, a resolution of 60,000 in the centroid mode with a mass range of *m*/*z* 50–1000 was used. 

### 2.9. Statistical Analysis and Metabolite Identification

The peaks from the chromatogram obtained from the LC-MS/MS were extracted using Compound Discoverer 2.0 (Thermo Fisher Scientific, Waltham, MA, USA). After normalization using the internal standard, the relative intensities of the components were obtained. The components were then analyzed by principal component analysis (PCA) using the statistical analysis provided by MetaboAnalyst 4.0 (www.metaboanalyst.ca) and Mass Fragment software (Waters, Milford, MA, USA). Using our in-house database together with online databases such as HMDB (http://www.hmdb.ca/), METLIN (http://metlin.scripps.edu/), KEGG (http://www.genome.jp/kegg/), and Lipidblast (http://fiehnlab.ucdavis.edu/projects/LipidBlast), the identity of the metabolites were confirmed. The Student’s *t*-test provided statistically significant metabolites among different groups. Metabolites showing significant changes were visualized by heatmap analysis using the MetaboAnalyst 4.0 [28]. 

## 3. Results

### 3.1. Effects of FAS Inhibitors on the Cell Viability and FAS Enzyme Activity of the Human Prostate Cancer LNCaP-LN3 Cell Line

To characterize the effects of FAS inhibitors on cancer cell growth and survival, studies were performed to assess the effect of FAS inhibition. We performed a WST-1 assay on LNCaP-LN3 cells with three drugs and found that the drugs inhibited cell proliferation in a concentration-dependent manner (Figure 1). The prostate cancer cell line was examined after treatment with different concentrations of Fasnall, GSK2194069, and TVB-3166. All three FAS inhibitors significantly reduced cell viability at 50 μM. FAS enzyme activity assay shows that GSK2194069 and TVB-3166 inhibit the human purified FAS activity with an IC_50_ of 0.0604, and 0.0736 μM, respectively. Fasnall inhibits the purified human FAS activity with an IC_50_ of 3.71 μM according to a recent study [25].

### 3.2. Metabolomics Changes Following Inhibition of FAS in Human LNCaP-LN3 Prostate Cancer Cells

To determine the effects of FAS inhibitors on the metabolite profile, we carried out metabolomic analysis by UPLC-Orbitrap-MS/MS following 24 h of exposure of LNCaP-LN3 cells to 50 µM FAS inhibitors. The QC samples were adapted to monitor and evaluate the stability of the analysis. In total, 20 cell extracts (5 parallel biological samples for each drug) and 5 QCs were analyzed. A total of 1265 and 733 features were detected in the positive and negative ion modes, respectively. A PCA (principal component analysis) was initially performed on the LC-MS datasets for each peak extracted from Compound Discoverer 2.0 to obtain the natural clustering trend for the four drugs in LNCaP-LN3 cells. As shown in Figure S1, apparent clustering and separation were observed among the drugs in both ion modes. We identified a total of 56 significant polar and nonpolar metabolites—including amino acids, carnitines, phospholipids, and sphingolipids—through the METLIN, HMDB, Lipidblast, and KEGG online databases and in-house databases.

**Figure 2:**
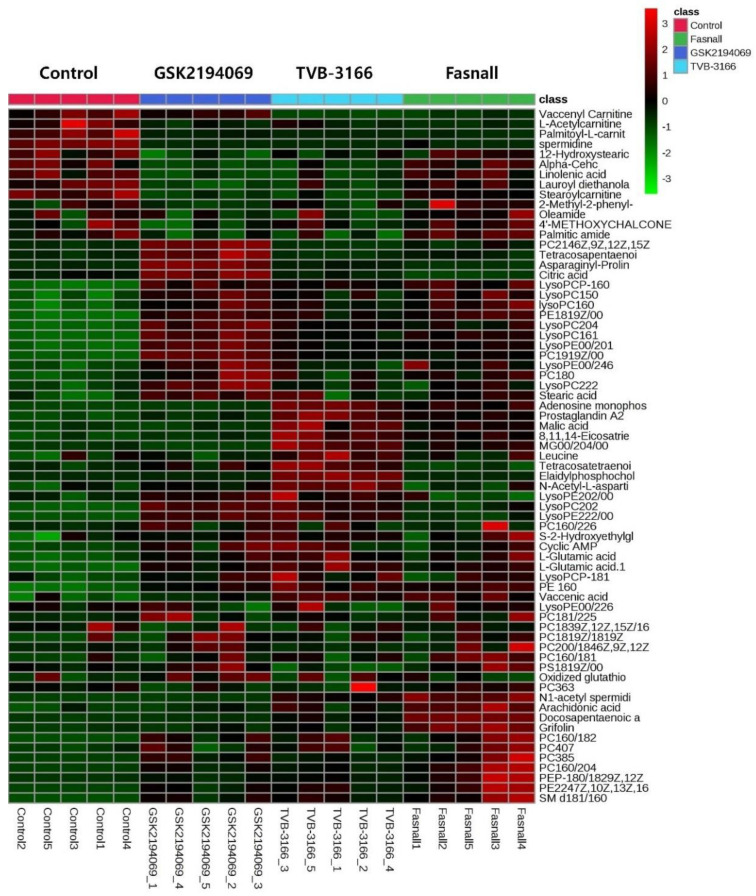
Hierarchical cluster analysis of identified metabolites. The metabolites were clustered, and shades of green and red represent downregulation or upregulation.

**Figure 3:**
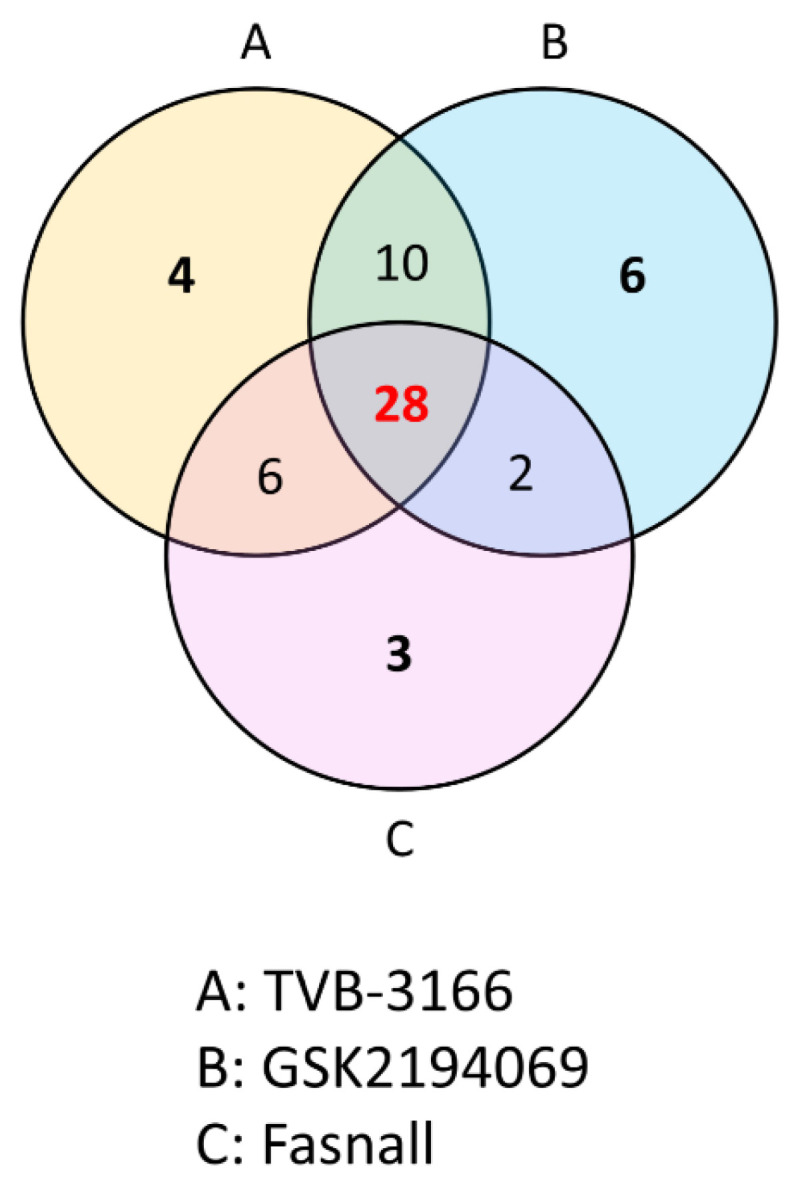
Venn diagram comparison analysis. An overlap of the common metabolites of the TVB-3166 (group A), GSK2194069 (group B), and Fasnall (group C) from the metabolite analysis.

As we have shown in the heatmap below, each group has distinctive features and is well distinguished (Figure 2). Appendix A lists the significantly changed metabolites after the treatment of FAS inhibitors. The carnitines such as L-acetyl carnitine, stearoyl carnitine, vaccenyl carnitine, and palmitoyl-L-carnitine were significantly decreased in the cells treated with FAS inhibitors. Sphingomyelin (d18:1/16:0) also showed a significant increase as much as 20-folds or more in the drugs treated group. Lipids including glycerophospholipids, PE(16:0), PE(18:1), LysoPE(20:1), LysoPE(24:6), PC(34:2), PC(36:4), PC(38:5), LysoPC(15:0), LysoPC(16:1), LysoPC(20:2), and LysoPC(16:0) were also significantly increased with the drugs. Glucose-6-phosphate levels were decreased while glutamate levels were increased in general after the FAS inhibitor treatment. Also, polyamine levels such as N1-acetylspermidine showed a significant increase while spermidine was significantly decreased in all of the groups. Figure 3 shows a Venn diagram illustrating the number of distinct and common metabolites among the three drug groups. 

### 3.3. Effects of Extracellular Palmitate on the Cell Viability during FAS Inhibition in Fatty Acid Free Media

**Figure 4:**
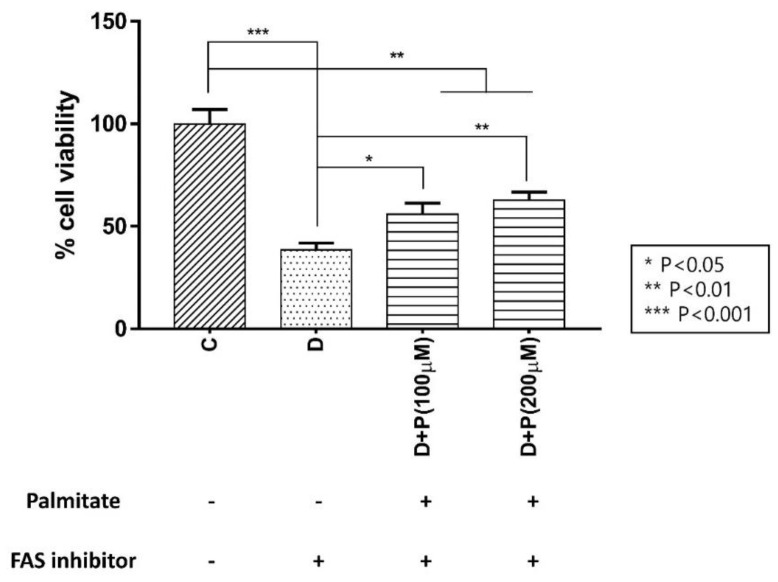
Effect of extracellular palmitate during FAS inhibitor treatment on LNCaP-LN3 human prostate cancer cell viability. (C: control group, D: TVB-3166 group, D + P (100 μM): TVB-3166 with 100 μM palmitate, D + P (200 μM): TVB-3166 with 200 μM palmitate).

Because FAS inhibition effectively prevented cell growth, we wanted to confirm if supplying the product of FAS, palmitate, can overturn this effect. We performed a CCK-8 assay on LNCaP-LN3 cells with TVB-3166 incubated together with extracellular palmitate and found that palmitate effectively rescued the cell proliferation in a concentration-dependent manner (Figure 4). 

### 3.4. Metabolomics Analysis of FAS Inhibition with Extracellular Palmitate 

To determine the effects of palmitate on the cellular metabolism during FAS inhibition, we carried out metabolomic analysis using UPLC-Orbitrap-MS/MS following 24 h of exposure of LNCaP-LN3 cells to 50 µM of TVB-3166 together with palmitate. The QC samples were adapted to monitor and evaluate the stability of the analysis. In total, 15 cell extracts (5 parallel biological samples for each group, C, D, D + P) and 5 QCs were analyzed. A total of 789 and 341 features were detected in the positive and negative ion modes, respectively. A principal component analysis (PCA) was initially performed on the LC-MS/MS datasets for each peak extracted from the software Compound Discoverer 2.0 to obtain the natural clustering trend for the three groups in LNCaP-LN3 cells. As shown in Figure S2, apparent clustering and separation were observed among the groups in both ion modes. As a control, the internalization of palmitate was confirmed by using isotopic palmitic acid-d_31_ and the peak indicating it was only found in D + P group (Figure S3). 

We identified a total of 56 significantly changed polar and nonpolar metabolites—including amino acids, carnitines, phospholipids, and sphingolipids—through the METLIN, HMDB, Lipidblast, and KEGG online databases together with our in-house database. 

**Figure 5:**
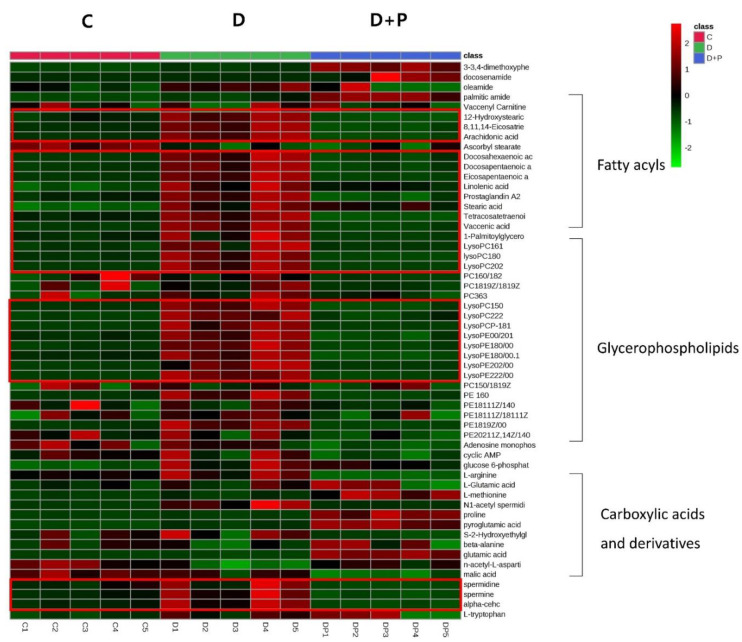
Hierarchical cluster analysis of identified metabolites. The metabolites were clustered, and shades of green and red represent downregulation or upregulation.

The heatmap below shows distinctive features within the group (Figure 5). The drug-treated D group showed distinctively different metabolite profiles from the control while the palmitate treated D + P group showed the metabolite profiles are similar to that of the control C group. 

Of the identified metabolites, 41 metabolites showed significant changes between D + P and D groups. Among the significantly changed metabolites, the ones with a *p*-value less than 0.001 were glutamic acid, LysoPE (20:1), vaccenic acid, LysoPC (15:0), pyroglutamic acid, eicosatrienoic acid, proline, and arachidonic acid.

Fatty acids including stearic acid, linolenic acid, arachidonic acid, tetracosapentaenoic acid, docosapentaenoic acid, and vaccenic acid were significantly increased after the FAS inhibitor treatment and generally returned to the level similar to the control group when palmitate was treated. Also lipids such as glycerin, phospholipids, LysoPC (15:0), LysoPC (16:1), lysoPC (18:0), LysoPC (20:2), LysoPC (22:2), LysoPC (P-18:1), LysoPE (20:1), LysoPE (18:0), LysoPE (20:2), LysoPE (22:2), PE (16:0), and PE (18:1(9Z)/0:0) were significantly increased only in the D group. Amino acid and biogenic amines such as arginine, spermidine, adenosine monophosphate, and malic acid were significantly decreased only in the drug-treated D group, while L-methionine, palmitic amide, glutamic acid, and proline were significantly increased only in the D + P group. In general, treating extracellular palmitate together with FAS inhibitor partially reversed many of the significantly changed metabolites back to its original state.

### 3.5. Oil-Red O Staining in a Fatty Acid Free Medium

**Figure 6:**
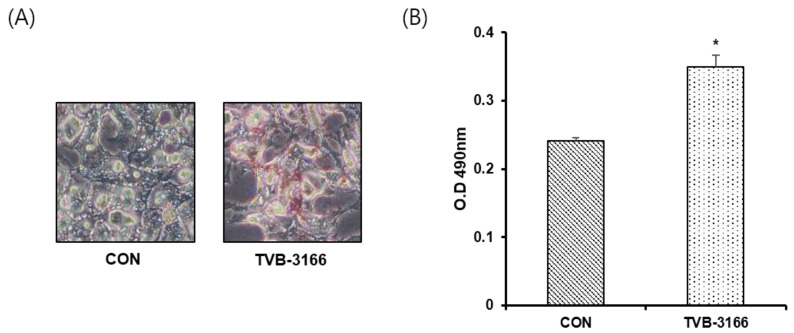
Quantitative Oil-Red O staining of LNCaP_LN3 cells. LNCaP_LN3 cells were treated with 50 μM of TVB-3166 in fatty acid-free media for 24 h, then stained using the optimized protocol (see Materials and Methods). (**A**) Representative images of untreated (CON) and TVB-3166 treated cells. (**B**) Quantitative evaluation of staining in untreated and TVB-3166 treated cells. *, *p* < 0.05 by Student’s *t*-test.

To confirm that increased glycerophospholipids detected after FAS inhibition is not due to extracellular lipid uptake, we have performed Oil-Red O staining of cells incubated in media containing fatty acid-free BSA. As shown in Figure 6, TVB-3166 treated cells showed significantly increased lipid droplet contents compared with untreated cells.

## 4. Discussion

Cancer cells are well known for their metabolically flexible nature, allowing them to survive under various metabolic stress [12]. FAS is an essential enzyme involved in de novo lipogenesis, synthesizing palmitate, a basic building block of long-chain fatty acids [29]. Here we describe the metabolic alteration of the prostate cancer cell line, LNCaP-LN3, during FAS inhibition with the second generation FAS inhibitors, TVB-3166, GSK2194069, and Fasnall. By limiting the availability of palmitate through inhibiting FAS in the cancer cell, we show significant alteration in the endogenous metabolite profiles including that of the lipids.

**Figure 7:**
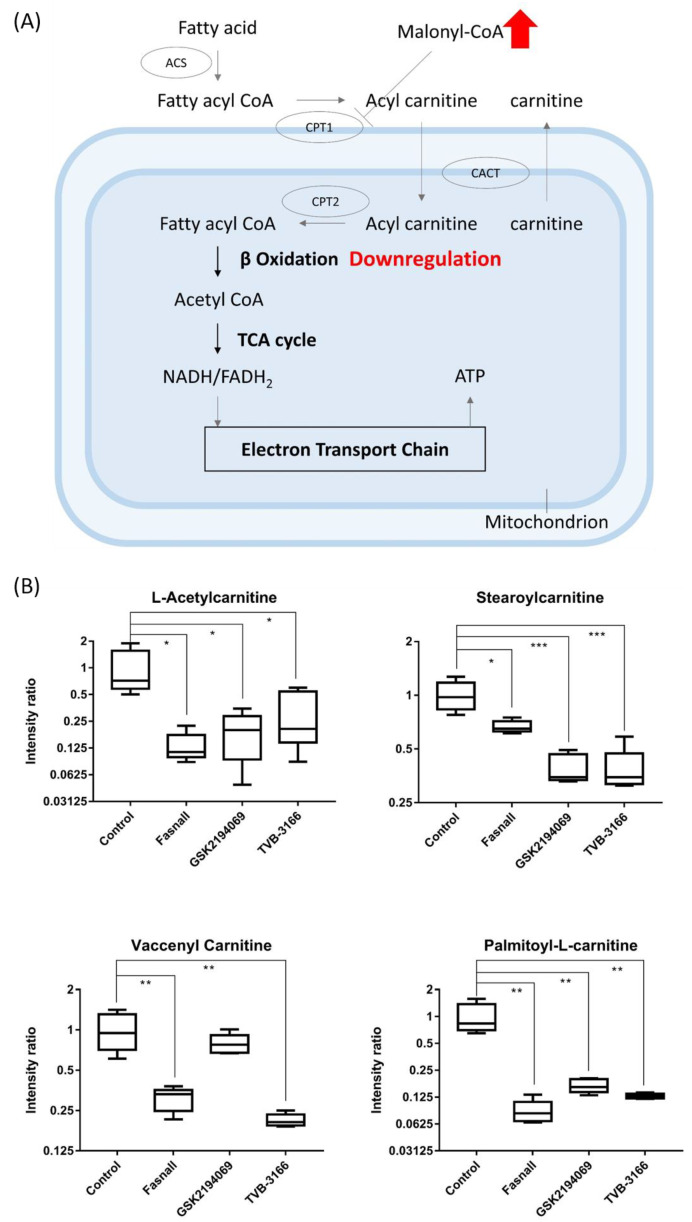
Carnitine box plot and beta-oxidation pathway. (**A**) Box plots showing the levels of carnitines from metabolomics data, (n = 5 per condition) *** *p* < 0.001, ** *p* < 0.01, * *p* < 0.05, Student’s *t*-test. (**B**) The metabolic pathway of fatty acid oxidation in which CPT-1 can be inhibited directly by malonyl-CoA, a crucial intermediate in FAS.

The accumulation of the substrates, acetyl-CoA and malonyl-CoA, during FAS inhibition may trigger the secondary effects. The first thing that we observed was the decreased levels of carnitines in the drug-treated groups. In addition to its role as a substrate of FAS, malonyl-CoA is used to regulate energy through the reversible inhibition of carnitine palmitoyltransferase-1 (CPT-1) [30]. The proteins of CPT-1 family (CPT-1A, CPT-1B, CPT-1C) is responsible for transporting long-chain fatty acids (FAs) from the cytoplasm into the mitochondria for oxidation and CPT-1A is involved in catalyzing the rate-limiting step of fatty acid oxidation [31]. Under the condition of excess energy, the fatty acid oxidation is prevented through the inhibition of CPT-1 by increased malonyl-CoA. Conversely, malonyl-CoA level falls to proceed with fatty acid oxidation to allow energy production during starvation (Figure 7A) [32]. 

The accumulation of the substrate malonyl-CoA would inhibit CPT-1 and thus limit the production of acylcarnitine and lead to the downregulation of fatty acid β-oxidation (Figure 7B). Proliferation and growth of cancer cells will also be suppressed as β-oxidation, an important metabolic pathway for energy production, is reduced [33]. Our results clearly show that acylcarnitines were significantly downregulated after the FAS inhibition (Figure 6A), suggesting that the FAS inhibitors may indirectly affecting the activity of CPT-1. 

**Figure 8:**
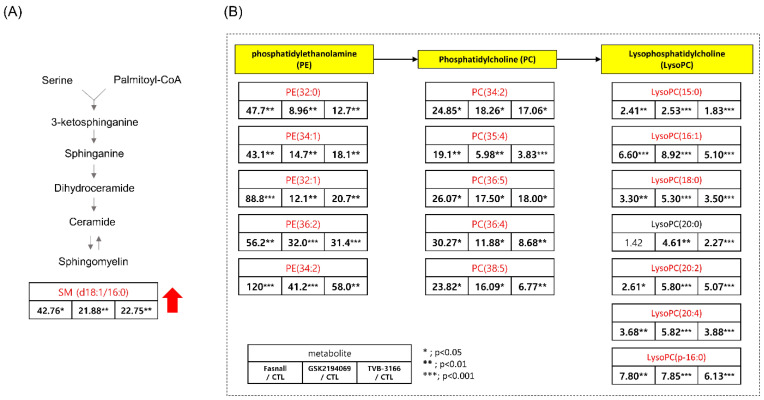
Table showing the fold change in SM(d18:1/16:0), PE, PC, and LysoPC in FAS inhibitors (**A**) Table showing the fold change in SM(d18:1/16:0) in the Fasnall-, GSK214069-, and TVB-3166-treated groups from metabolomics data (n = 5 per condition), *** *p* < 0.001, ** *p* < 0.01, * *p* < 0.05, Student’s *t*-test. (**B**) Table showing the fold change in the PE, PC, and LysoPC family in the Fasnall-, GSK214069-, and TVB-3166-treated groups from metabolomics data (n = 5 per condition), *** *p* < 0.001, ** *p* < 0.01, * *p* < 0.05, Student’s *t*-test. Metabolites in red are the commonly increased metabolites only with Fasnall, GSK2194069, and TVB-3166 drug treatment.

Besides, dodecanoic acid produced by oxidation from palmitate was reduced to 0.063-, 0.036-, and 0.032-fold in the Fasnall, GSK2194069, and TVB-3166 group, respectively (Appendix A). Treating with the drugs has significantly increased the level of sphingomyelin (SM; d18:1/16:0), phosphosphingolipids composed of oleic acid on the C1 site and palmitic acid on the C2 site, by more than 20-fold (Figure 8A). Because CPT-1 uses fatty acyl-CoA to produce acylcarnitine, the inhibition of CPT-1 may cause an accumulation of palmitoyl-CoA, which is then used for the production of SM (d18:1/16:0) [34]. C16 sphingomyelin, a metabolite known to be accumulated in prostate cancer tissues, is one of the biomarkers that is predictive of prostate cancer aggression [35]. However, the increased levels of ceramide and sphingomyelin are some of the known distinct features of apoptotic signaling in cancer cells concerning lipid metabolism [36,37,38]. Interestingly, our results showed that sphingomyelin levels were increased in the Fasnall-, GSK2694069-, and TVB-3166-treated groups, suggesting that CPT-1 modulation may have caused these effects.

**Figure 9:**
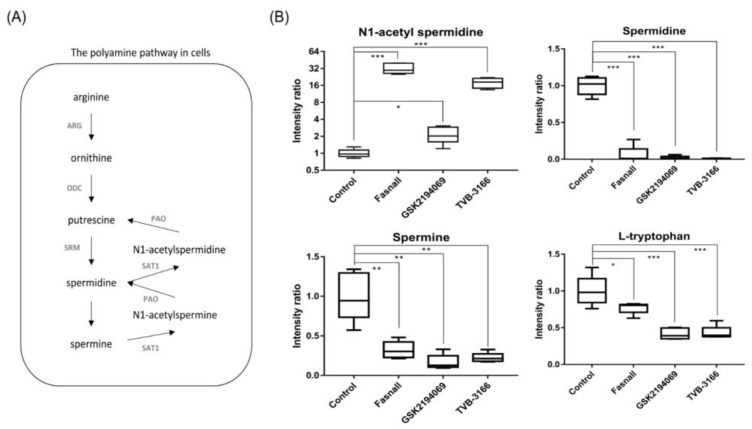
Polyamine pathway and box plots showing the fold change in the polyamine pathway metabolites. (**A**) The polyamine pathway shows metabolites involved in polyamine metabolism. (**B**) Box plots show the relative intensity ratio of polyamine metabolites and tryptophan with FAS inhibitors, (n = 5 per condition), *** *p* < 0.001, ** *p* < 0.01, * *p* < 0.05, Student’s *t*-test.

Significant changes in the metabolites in the polyamine metabolism (Figure 9A) were observed after the treatment with FAS inhibitors. In particular, the levels of N1-acetyl spermidine were significantly increased while the levels of spermidine, spermine, and tryptophan were significantly decreased in the Fasnall, GSK2194069, and TVB-3166 groups (Figure 9B). Polyamine is a small organic cation that is essential for normal cell growth and development and plays an important role in ion channel regulation when cancer cells grow, and the activity of each enzyme is linked to lipid polyamine metabolism by changes in acetyl-CoA and ATP. It is known that polyamine metabolism is dysregulated in tumors, with an increase in polyamine levels, unlike in the normal state [39]. 

N1-acetylspermidine is known to exist in a larger amount in tumor tissues, such as breast cancer and colorectal cancer, than in normal tissues due to an increase in the activity of spermidine/spermine N1-acetyltransferase (SAT1) [40,41]. Since acetyl-CoA is expected to accumulate due to FAS inhibition, spermidine and spermine may become acetylated, and N1-acetylspermidine and N1-acetylspermine are expected to increase [42]. As expected, we found that N1-acetyl spermidine was significantly increased, while spermidine and spermine were significantly decreased. The strictly regulated polyamine concentration in the steady-state is unmaintained when acetyl-CoA is actively acetylating polyamines, which will have various effects, such as failing to stabilize the structure of the cancer cell, controlling the ion channel, and maintaining the membrane stability [43]. The increased level of N1-acetylspermidine in our results suggests that polyamine metabolism in the LNCaP-LN3 prostate cancer cells may have been dysregulated due to the increased level of acetyl-CoA. The increase in N1-acetylspermidine may have metabolic consequences that lead to cell survival [44]. N1-acetylspermidine has a greater effect on the production of putrescine compared to the effect on the production of ornithine [45]. The putrescine produced from the N1-acetylspermidine is used as an instant energy source by the cells, and it will maintain the growth of cancer cells [46,47]. Putrescine may also increase the survival of cancer cells exposed to oxidative stress [44].

**Figure 10:**
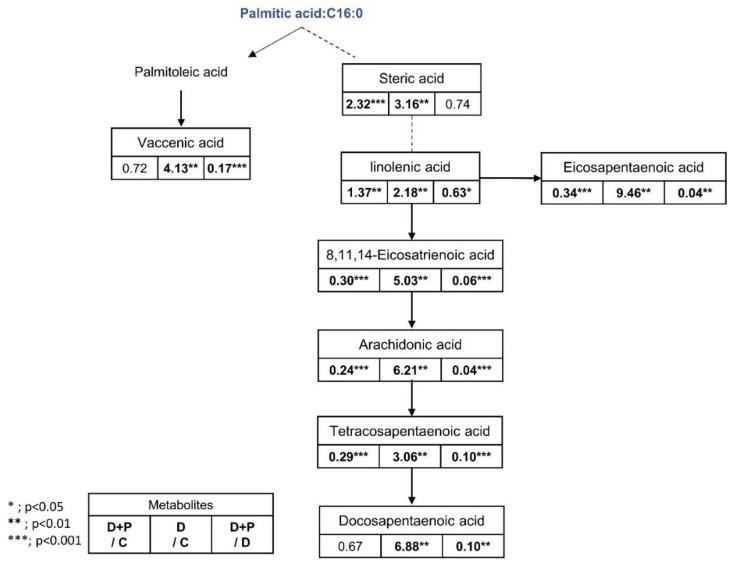
Fatty acid pathway and fold change table. Table showing the fold change in fatty acids in the D + P-, D, and D + P-treated groups from metabolomics data (n = 5 per condition), *** *p* < 0.001, ** *p* < 0.01, * *p* < 0.05, Student’s *t*-test.

However, the most prominent alteration in the metabolite levels was observed in the lipid metabolism. Glycerophospholipids (PE, PC, and LysoPC) were found to be increased in all three drug groups (Figure 8B). The increased levels of glycerophospholipids and fatty acids such as arachidonic acid (Figure 10) during FAS inhibition were most unexpected results because de novo lipogenesis was inhibited and it was expected that the lipid levels to fall in general. There may be several reasons for this phenomena. Tumor cells that are highly proliferative needs to synthesize de novo fatty acids to continually provide glycerophospholipids for membrane production. Increased fatty acid production also plays an important role in tumor cell survival and affects basic cellular processes, including signal transduction and gene expression [48]. Thus, the glycerophospholipids increased in our data may suggest an effort taken by the cancer cells to provide needed material for cell survival during the limited palmitate availability. However, the question still remains regarding the source of the increased lipids. 

We have incubated the cells with the serum-free and fatty acid-free media with and without palmitic acid to examine the source of the increased lipid during FAS inhibition. First, we have determined the effects of external palmitic acid on the cell viability when incubated with FAS inhibitors. As shown in Figure 4, the D + P (a drug with palmitate, in serum-free media) group showed a significant increase in cell viability as palmitate concentration increased compared to the D (drug, in serum-free media) group. However, the recovery was not complete with the external palmitate supplementation. The accumulated malonyl-coA may be inhibiting CPT-1 enzyme as an indirect effect of FAS inhibition, causing downregulation of fatty acid β-oxidation. Therefore, the FAS inhibited cells supplemented with exogenous palmitate can only be partially recovered. 

**Figure 11:**
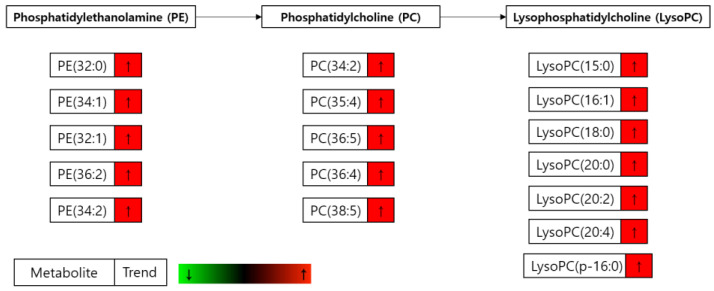
Significantly changed glycerophospholipids. Changes in the glycerophospholipid levels in the drug-treated cells compared to the drug plus palmitate treated cells (D group/D + P group) incubated in the serum-free media.

It is intriguing that even in the fatty acid and lipid free condition, the cells treated with FAS inhibitor showed significantly increased levels of fatty acids (Figure 10) and glycerophospholipids (Figure 11). These results suggest that the increased lipids after FAS inhibition were endogenously regulated. To support the idea, we have performed Oil-Red O lipid staining experiment and were able to confirm significantly higher levels of lipids in the cells treated with FAS inhibitor. 

Two of the possibilities by which glycerophospholipids can be synthesized and regulated in the cells are the Kennedy pathway and the Lands cycle. The Kennedy pathway involves CDP-choline pathway and the CDP-ethanolamine pathway that produces PC and PE from choline and ethanolamine, respectively [49]. Since PEs and PCs are important building blocks for the phospholipid bilayer and can influence energy metabolism, to compensate the energy loss through the downregulation of β-oxidation, the Kennedy pathway may have been activated [50,51]. Choline uptake into the cells may have taken place through choline transporter (ChT) or through choline transporter-like protein 1 (CTL1) which are known to be overexpressed in LNCaP-LN3 cells [52,53]. The Lands cycle involves the formation of LysoPC from PC by the enzyme, phospholipase A_2_ (PLA_2_), through removing fatty acids at the *sn-2* position [54]. The fatty acids released from the Lands cycle is frequently arachidonic acid [55]. Thus, our results showing a significant increase in LysoPC, LysoPE, and fatty acids level after FAS inhibition may be due to the regulation of the PLA_2_ enzymes in the Lands cycle. 

Another possibility may be the lipid droplets involved in modulating lipid metabolism [56]. Lipid droplets mostly store triacylglycerol (TAG) but also stores polyunsaturated FAs such as arachidonic acid, which are the precursors of eicosanoids [57]. Also, in a recent study, lipid droplets were found to be present in the nucleus and could regulate PC synthesis [58]. Thus, in an event of FAS inhibition, the blockade of de novo lipogenesis may trigger TAG breakdown in the lipid droplets and could release fatty acids and diacylglycerol (DAG).

Furthermore, several studies reported that CPT-1 inhibition effectively increased the levels of glycerophospholipids [48,59,60]. Recently, when mice were treated with etomoxir, an inhibitor of CPT-1, increased circulating FA and triacylglycerol accumulation in the liver and heart were observed within a few hours [60]. Since our study also implies the inhibition of CPT-1 due to the accumulation of malonyl-CoA, the accumulation of the glycerophospholipids in our data may implicate the CPT-1 inhibition as a secondary effect associated with the inhibition of FAS.

**Figure 12:**
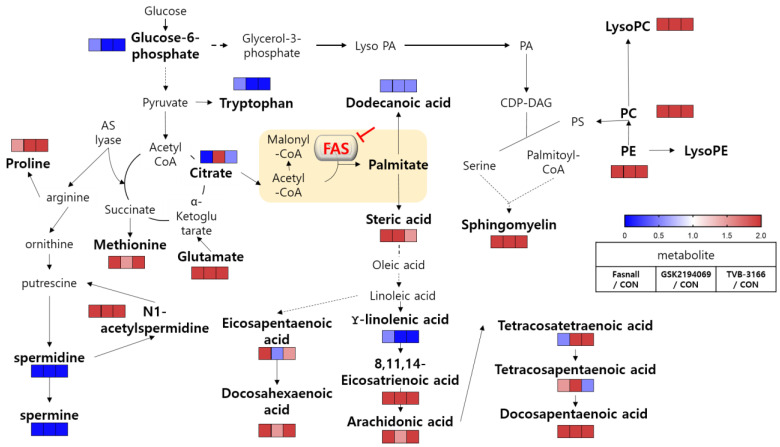
Overview of metabolic pathway analysis. Blue color boxes indicate a decrease in compounds and metabolic pathways, while red boxes indicate an increase. Bold indicates significantly changed metabolites.

Drugs that have the same enzyme target may perform at the cellular level due to various unknown effects, such as off-target effects, drug resistance, and metabolic flexibility. We evaluated three drugs that target FAS, a therapeutic target for anticancer drugs, using metabolomics. All three drugs showed similar potency in the cellular viability assays, but interestingly, the metabolite changes within the cells were not all the same with these drugs. The overall change in the metabolic pathway was analyzed and illustrated in Figure 12. In general, FAS inhibitors caused the levels of carnitine and dodecanoic acid to fall, indicating that β-oxidation, which causes lipid hydrolysis, is downregulated possibly by the accumulation of malonyl-CoA. Various metabolites in the polyamine pathway were also significantly changed. The levels of spermidine and spermine were significantly decreased, while N1-acetylspermidine was increased, probably due to the accumulation of acetyl-CoA. Interestingly, and unexpectedly, long-chain unsaturated fatty acids and glycerophospholipids were increased when all three FAS inhibitors were used. According to our study results, the increase in fatty acids is not the result of the absorption of extracellular lipids, but it was rather from an endogenous synthetic pathway which may be the aftermath of metabolic reprogramming. The possible mechanistic explanations are provided, however the exact mechanism of increased lipid molecules in an event of FAS inhibition needs to be elucidated.

To our knowledge, this is the first time that these three drugs were compared using a metabolomics study, and we believe that our results offer some important information regarding metabolites involved in these drugs’ secondary effects at the cellular level. Furthermore, our proposed results regarding the metabolic alterations from each drug may provide important insights for future drug development targeting cancer metabolism.

## Supplementary Materials

The following are available online at https://www.mdpi.com/2073-4409/9/11/2447/s1, Figure S1: The 3D score plots of PCA from the metabolomic analysis of treated LNCaP-LN3 cells with FAS inhibitors, Figure S2: The 3D score plots of PCA from the metabolomic analysis of treated LNCaP-LN3 cells with external palmitate, Figure S3: palmitic acid-d_31_ peak in UPLC-Orbitrap MS/MS.

## Appendix A

**Table A1:** Metabolites altered significantly after the drug treatment. Student’s *t*-test was performed to evaluate the statistical significance of changes in metabolite levels. Bold indicates that the changed metabolites are statistically significant. (↑: upward trend, ↓: downward trend).

Metabolite	MW	Fasnall			GSK2194069			TVB-3166		
		*p*-Value	Fold Change	Trend	*p*-Value	Fold Change	Trend	*p*-Value	Fold Change	Trend
12-Hydroxystearic acid	300.26	0.278	0.83		**0.002**	0.42	↓	**0.019**	0.62	↓
8,11,14-Eicosatrienoic acid	306.25	**<0.000**	3.73	↑	**0.023**	1.72	↑	**<0.000**	5.97	↑
Acetylcarnitine	203.12	**0.027**	0.13	↓	**0.032**	0.19	↓	**0.036**	0.32	↓
Adenosine monophosphate	347.06	**0.001**	17.03	↑	**0.010**	3.04	↑	**<0.000**	27.34	↑
Alpha-Cehc	278.15	0.570	0.93		**0.005**	0.47	↓	**0.005**	0.55	↓
Arachidonic acid	304.24	**<0.000**	3.41		0.151	1.33		**0.025**	2.00	↑
Asparaginyl-Proline	229.09	**0.006**	0.69		**<0.000**	6.12	↑	**0.003**	1.44	↑
Citric acid	192.03	**<0.000**	0.21	↓	**<0.000**	3.21	↑	0.058	0.77	
Cyclic AMP	329.07	0.055	2.04		**0.021**	2.86	↑	**0.044**	2.81	↑
Docosapentaenoic acid	330.25	**<0.000**	6.66	↑	**0.015**	1.23	↑	**0.006**	2.65	↑
Elaidylphosphocholine	433.33	**0.032**	1.45	↑	**0.002**	1.68	↑	**<0.000**	30.41	↑
Glutamic acid	147.05	**0.001**	4.45	↑	**<0.000**	4.30	↑	**0.001**	6.11	↑
Grifolin	328.24	**<0.000**	2.70	↑	0.190	1.14		**0.008**	1.36	↑
Lauroyl diethanolamide	287.25	0.124	0.76		**<0.000**	0.28	↓	**0.008**	0.45	↓
Leucine	131.07	0.393	1.17		0.965	1.01		**0.011**	1.64	↑
Linolenic acid	278.19	0.517	0.92		**0.001**	0.47	↓	**0.007**	0.56	↓
LysoPC(15:0)	481.31	**0.001**	2.41	↑	**<0.000**	2.53	↑	**0.009**	1.83	↑
LysoPC(16:0)	495.33	**<0.000**	2.27	↑	**0.001**	2.18	↑	**0.002**	1.76	↑
LysoPC(16:1)	493.32	**<0.000**	6.60	↑	**<0.000**	8.92	↑	**<0.000**	5.10	↑
LysoPC(20:2)	547.36	**0.011**	2.61	↑	**<0.000**	5.80	↑	**<0.000**	5.07	↑
LysoPC(20:4)	543.33	**0.002**	3.68	↑	**<0.000**	5.82	↑	**<0.000**	3.88	↑
LysoPC(22:2)	635.34	0.075	1.35		**<0.000**	2.04	↑	0.064	1.27	
LysoPC(P-16:0)	539.32	**0.001**	7.80	↑	**<0.000**	7.85	↑	**<0.000**	6.13	↑
LysoPC(P-18:1)	565.33	0.116	1.56		**0.011**	1.74	↑	**0.023**	2.15	↑
LysoPE(0:0/20:1)	567.35	**<0.000**	3.19	↑	**<0.000**	4.56	↑	**<0.000**	2.93	↑
LysoPE(0:0/24:6)	613.33	**0.018**	1.83	↑	**<0.000**	2.14	↑	**0.048**	1.37	↑
LysoPE(20:2/0:0)	505.31	0.657	0.92		**0.001**	1.48	↑	**0.020**	1.59	↑
LysoPE(22:2/0:0)	593.37	**0.007**	2.27	↑	**<0.000**	6.04	↑	**0.001**	6.00	↑
Malic acid	134.02	**<0.000**	2.69	↑	0.641	1.09		**0.004**	4.67	↑
MG(0:0/20:4/0:0)	378.27	**0.001**	2.97	↑	**0.028**	0.63	↓	**0.001**	5.61	↑
N1-acetyl spermidine	187.17	**0.001**	32.29	↑	**0.019**	2.20	↑	**<0.000**	17.89	↑
N-Acetyl-L-aspartic acid	175.05	0.646	1.09		**0.033**	1.34	↑	**<0.000**	2.14	↑
Palmitic amide	255.26	0.194	1.18		**0.029**	0.64	↓	0.068	0.64	
Palmitoyl-L-carnitine	399.33	**0.006**	0.09	↓	**0.008**	0.17	↓	**0.007**	0.13	↓
PC(16:0/18:1)	759.58	**0.033**	2.62	↑	0.409	1.53		0.270	1.55	
PC(16:0/18:2)	757.56	**0.030**	24.85	↑	**0.012**	18.26	↑	**0.028**	17.06	↑
PC(16:0/20:4)	781.56	**0.015**	30.27	↑	**0.012**	11.88	↑	**0.004**	8.68	↑
PC(18:0)	523.36	**0.001**	2.40	↑	**0.014**	3.01	↑	0.071	1.86	
PC(19:1(9Z)/0:0)	595.38	**<0.000**	4.97	↑	**<0.000**	9.31	↑	**<0.000**	5.06	↑
PC(21:4(6Z,9Z,12Z,15Z)/0:0)	617.36	0.232	1.38		**<0.000**	4.46	↑	**0.023**	0.79	↓
PC(38:5)	807.58	**0.042**	23.82	↑	**0.010**	16.09	↑	**0.008**	6.77	↑
PE (16:0)	453.28	**<0.000**	3.22	↑	**<0.000**	2.74	↑	**<0.000**	3.44	↑
PE(18:1(9Z)/0:0)	479.30	**<0.000**	3.24	↑	**<0.000**	3.29	↑	**0.001**	2.64	↑
PE(22:4(7Z,10Z,13Z,16Z)/15:0)	753.53	**0.023**	73.19	↑	**0.018**	28.26	↑	**0.013**	34.66	↑
PE(P-18:0/18:2(9Z,12Z))	727.51	**0.025**	32.51	↑	**0.021**	6.65	↑	**0.005**	9.13	↑
Prostaglandin A2	334.29	**<0.000**	3.97	↑	**0.004**	2.02	↑	**<0.000**	6.86	↑
PS(18:1(9Z)/0:0)	523.29	0.171	1.49		0.120	1.48		**0.006**	0.50	↓
S-(2-Hydroxyethyl)glutathione	351.05	0.182	1.45		0.135	1.36		**0.050**	1.48	↑
SM (d18:1/16:0)	702.57	**0.016**	42.76	↑	**0.009**	21.88	↑	**0.004**	22.75	↑
spermidine	145.16	**<0.000**	0.06	↓	**<0.000**	0.02	↓	**<0.000**	0.01	↓
Stearic acid	284.27	**0.008**	1.45	↑	**<0.000**	1.76	↑	0.119	1.41	
Stearoylcarnitine	426.37	**0.015**	0.67	↓	**<0.000**	0.39	↓	**<0.000**	0.39	↓
Tetracosapentaenoic acid	358.28	0.241	1.16		**0.001**	3.59	↑	0.067	0.83	
Tetracosatetraenoic acid (24:4n-6)	360.30	0.081	0.82		**0.021**	1.48	↑	**0.002**	2.29	↑
Vaccenic acid	282.25	**0.003**	2.36	↑	0.128	1.46		**0.001**	2.39	↑
Vaccenyl Carnitine	425.35	**0.007**	0.31	↓	0.215	0.79		**0.005**	0.21	↓

Abbreviation: PC: phosphatidylcholine, PE: phosphatidylethanolamine, PS: phosphatidylserine, SM: sphingomyelin
