# Supplementary material for: Deciphering Fatty Acid Synthase Inhibition-Triggered Metabolic Flexibility in Prostate Cancer Cells through Untargeted Metabolomics
Source: Cells. 2020 Nov 10;9(11):2447. doi: 10.3390/cells9112447 (PMC7697567; doi:10.3390/cells9112447)
Supplement: Supplementary file 1 [file cells-09-02447-s001.pdf]

<Supplemental Information>

Deciphering Fatty Acid Synthase Inhibition-Triggered Metabolic Flexibility in Prostate Cancer Cells through Untargeted Metabolomics

Ju Eun Oh [1,2], Byung Hwa Jung [1,3], Jinyoung Park [1], Soosung Kang [2], and Hyunbeom Lee [1,4,*]

[1] Molecular Recognition Research Center, Korea Institute of Science and Technology, Seoul 02792, Korea; dhwndsm@naver.com (J.E.O.); jbhluck@kist.re.kr (B.H.J.); jypark@kist.re.kr (J.P.); hyunbeom@kist.re.kr (H.L.)

[2] College of Pharmacy and Graduate School of Pharmaceutical Sciences, Ewha Womans University, Seoul 03760, Korea; sskang@ewha.ac.kr (S.K.)

[3] Division of Bio-Medical Science &Technology, KIST School, Korea University of Science and Technology, Seoul 02792, Korea

[4] Department of HY-KIST Bio-convergence, Hanyang University, Seoul 04763, Republic of Korea

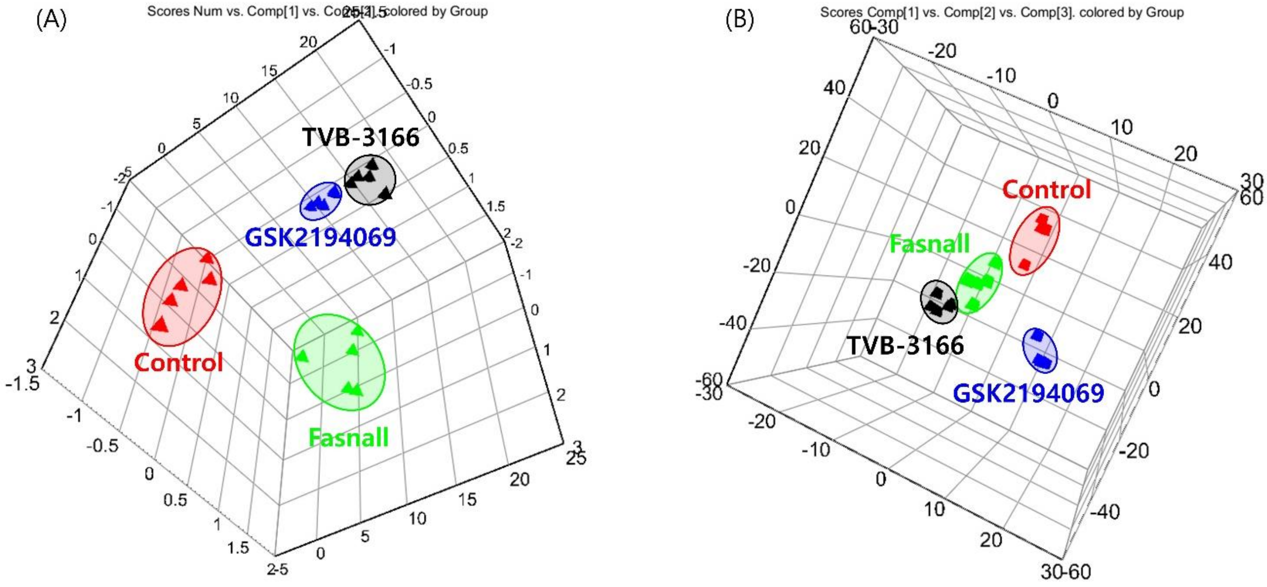

Figure S1. The 3D score plots of PCA from the metabolomic analysis of treated LNCaP-LN3 cells. Multivariate analysis using PCA was performed on data obtained from both positive A) and negative B) ionization modes.

<Supplemental Information>

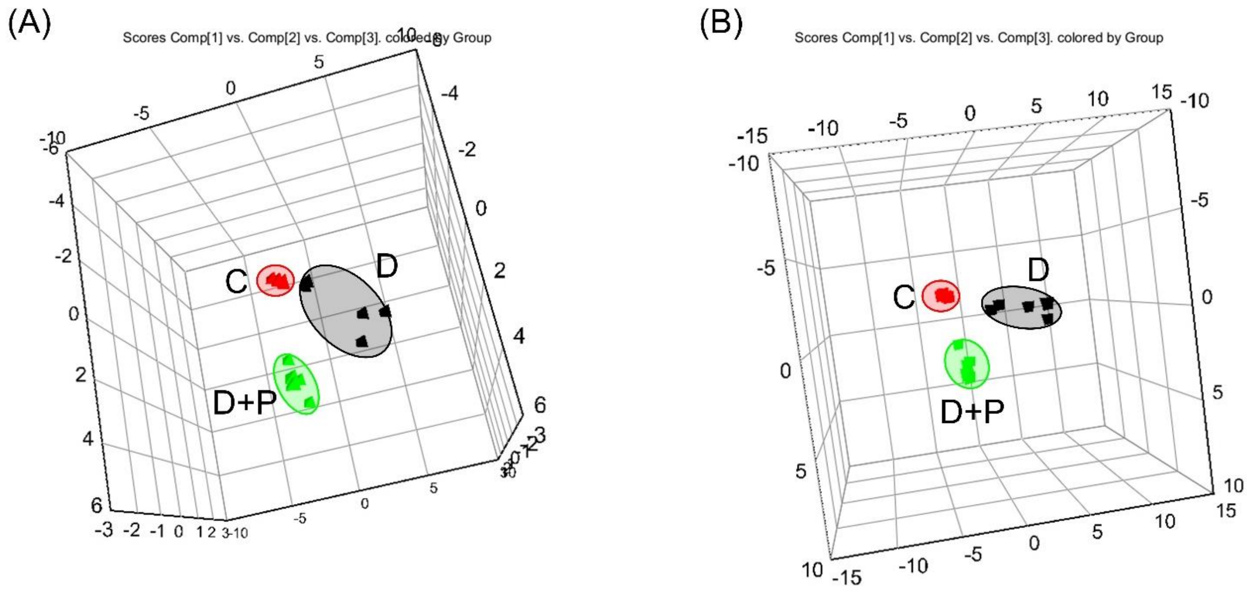

Figure S2. The 3D score plots of PCA from the metabolomic analysis of treated LNCaP-LN3 cells. Multivariate analysis using PCA was performed on data obtained from both positive A) and negative B) ionization modes. C is control, D is drug, ‘D + P’ is palmitate treated groups.

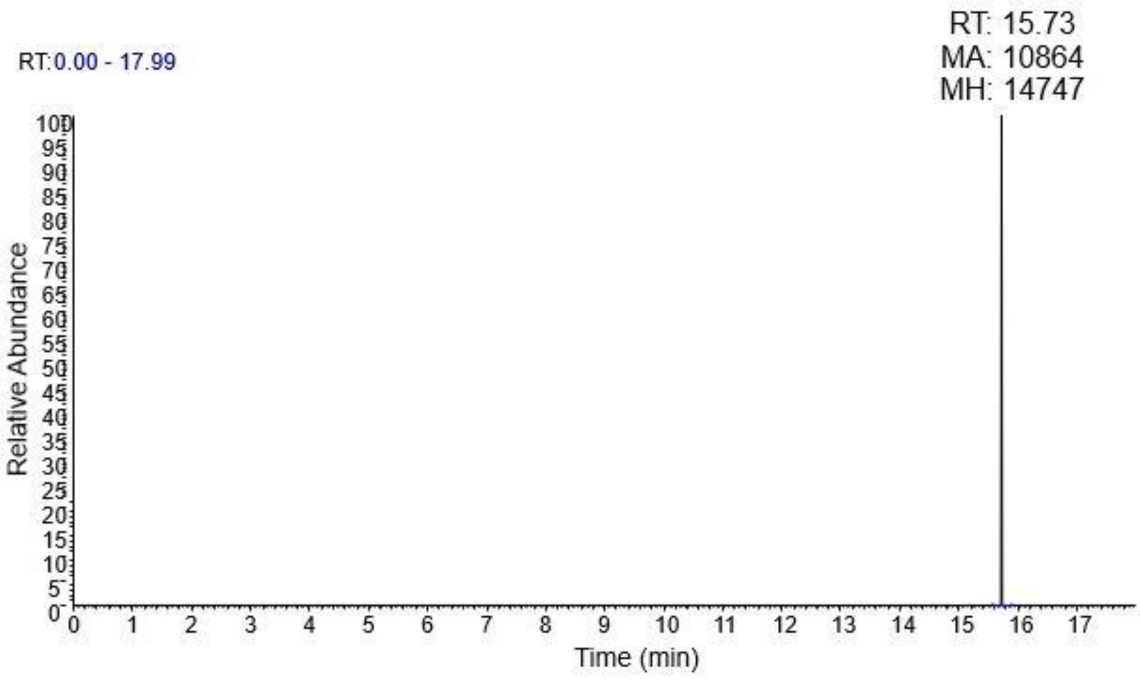

Figure S3. Palmitic acid-d_{31} peak in UPLC-Orbitrap MS/MS. Palmitic acid-d31 peaks was observed only in the palmitate treated groups.
